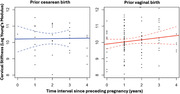# Oral Concurrent Session 3 – Ultrasound and Genetics: Thursday, January 30, 2025 1:30 PM – 4:00 PM

**DOI:** 10.1002/pmf2.12008

**Published:** 2025-01-05

## 29 | Beyond the Fetus: Unveiling Maternal Health Risks Through Prenatal Genome Sequencing

Brittany Arditi^1^; Caitlin D. Baptiste^2^; Jessica L. Giordano^1^; Rachel Herz‐Roiphe^2^; Vaidehi Jobanputra^3^; Ronald J. Wapner^4^

*^1^Columbia University Irving Medical Center, New York, NY; ^2^Columbia University Medical Center, New York, NY; ^3^New York Genome Center, New York, NY; ^4^Columbia University, New York, NY*

1:30 PM ‐ 1:45 PM

**Objective**: Genome sequencing (GS) has been demonstrated to be of benefit in the evaluation and management of fetal structural anomalies. However, the management of maternal ancillary findings on trio analysis has not been described. Our objective was to evaluate the frequency and clinical impact of maternal genomic variants identified after fetal GS.

**Study Design**: This is a secondary analysis of all patients undergoing prenatal GS between September 2022‐November 2023 at a single institution under a research protocol. 420 fetuses (279 anomalous and 141 non‐anomalous) were included. GS was performed in a CLIA certified laboratory and results reported to parents. Genome analysis was performed through a proband‐focused trio approach: pathogenic and likely pathogenic variants were identified in the fetus and parental inheritance was subsequently determined. All maternally inherited findings were adjudicated by a multidisciplinary team of maternal fetal medicine physicians, laboratory geneticists, and genetic counselors to determine the impact on pregnancy and postpartum care. Pathogenic and likely pathogenic variants in genes with implications for peripartum management were reported to the care providers.

**Results**: 3.8% (16/420) of patients had a maternally inherited, reportable, autosomal dominant finding. Among patients with a reportable finding, 9/16 (56.25%) were referred to a subspecialist for further management and/or underwent additional workup aimed at identifying and preventing risk for maternal morbidity (Table 1). Familial cascade testing was discussed with all patients and performed in 4/16 (25.0%) families. Only 2/16 (12.5%) of genes reported in our analysis are on the American College of Medical Genetics and Genomics secondary findings list.

**Conclusion**: In addition to providing a fetal diagnosis, prenatal sequencing will identify maternal genetic diseases with the potential to cause peripartum maternal morbidity and mortality. Future research is required to understand the clinical impact of these findings and to develop management protocols.

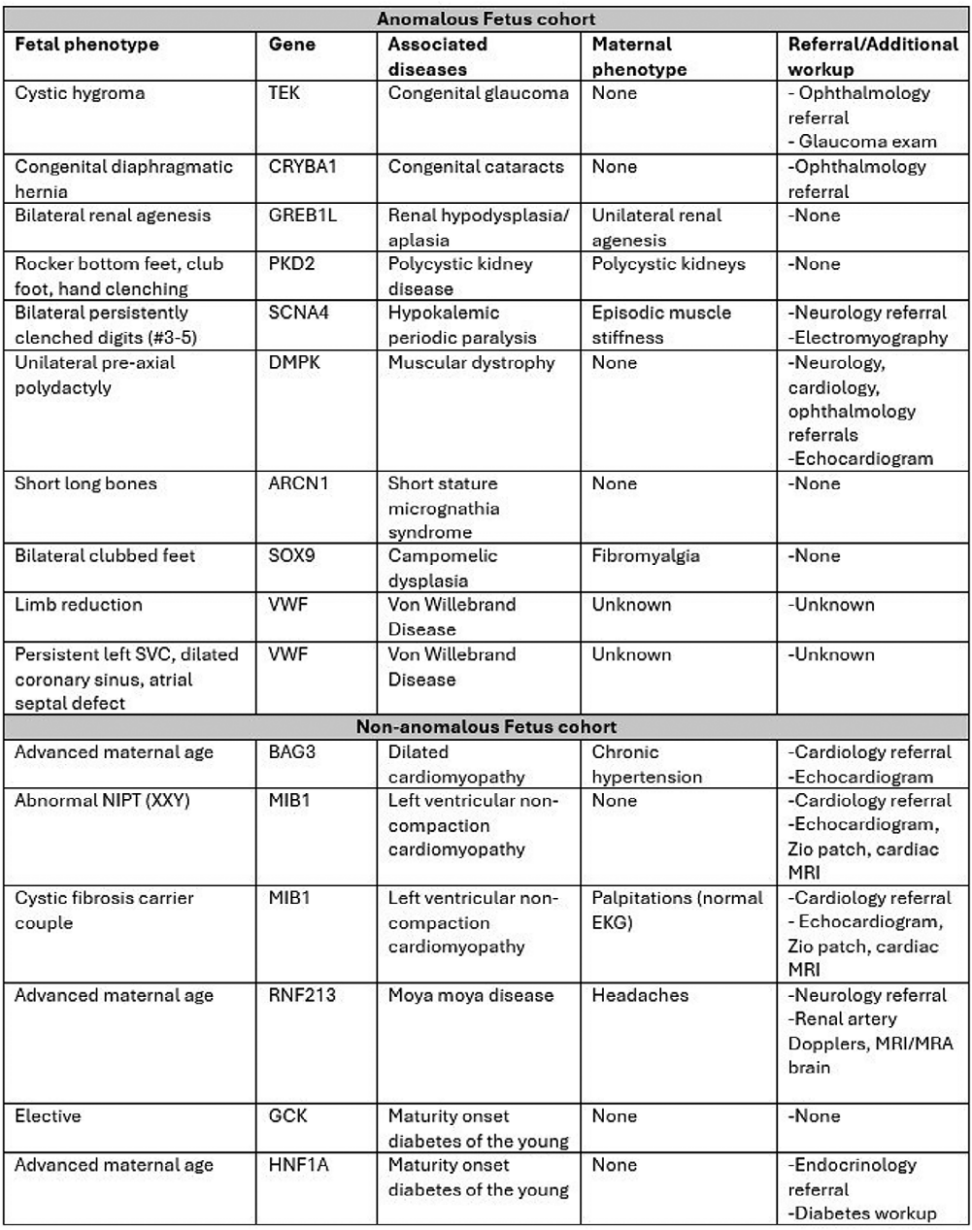

## 30 | Artificial Intelligence System Accurately Detects Fetal Ultrasound Findings Suspicious For Major Congenital Heart Defects

Carolyn M. Zelop^1^; Jennifer Lam‐Rachlin^2^; Alisa Arunamata^3^; Rajesh Punn^3^; Sarina K. Behera^4^; Matthias Lachaud^5^; Nadine David^6^; Greggory R. DeVore^7^; Andrei Rebarber^2^; Nathan S. Fox^2^; Marjorie Gayanilo^2^; Sara Garmel^8^; Philippe Boukobza^9^; Pierre Uzan^10^; Hervé Joly^11^; Romain Girardot^12^; Laurence Cohen^13^; Marilyne Levy^14^; Bertrand Stos^14^; Malo De Boisredon^15^; Eric Askinazi^15^; Valentin Thorey^15^; Christophe Gardella^15^; Miwa Geiger^2^

*^1^Valley Health System, Paramus, NJ; ^2^Icahn School of Medicine at Mount Sinai Hospital, New York, NY; ^3^Pediatrics ‐ Cardiology, Stanford University School of Medicine, Stanford, CA; ^4^Palo Alto Medical Foundation, Sutter Health, Palo Alto, CA; ^5^University of Grenoble Alpes, Grenoble, Rhone‐Alpes; ^6^Medical Training Center, Rouen, Haute‐Normandie; ^7^The Fetal Diagnostic Center of Pasadena, Pasadena, CA; ^8^Michigan Perinatal Associates and Corewell Health, Dearborn, MI; ^9^CEDEF ‐ Centre Européen de Diagnostic et d'Exploration de la Femme, Le Chesnay, Ile‐de‐France; ^10^Groupe IMEF ‐ Imagerie Médicale de l'Est Francilien, Rosny‐sous‐Bois, Ile‐de‐France; ^11^CARPEDIOL ‐ Cardiologie Pédiatrique, fœtale et congénitale adulte de L'Ouest Lyonnais, Ecully, Rhone‐Alpes; ^12^Unité de Dépistage de Cardiopathies Foetales et Néonatales, Bordeaux, Aquitaine; ^13^ETCC ‐ Exploration et Traitement des Cardiopathies Congénitales, Massy, Ile‐de‐France; ^14^UE3C ‐ Unité d'explorations cardiologiques ‐ Cardiopathies Congénitales, Paris, Ile‐de‐France; ^15^BrightHeart, Paris, Ile‐de‐France*

1:45 PM ‐ 2:00 PM

**Objective**: Prenatal detection of congenital heart defects (CHD) improves patient outcomes. Despite advances in imaging techniques and equipment, prenatal diagnosis remains low. We evaluated the accuracy of an artificial intelligence (AI) system to detect 2nd trimester (2T) ultrasound studies suspicious for CHD.

**Study Design**: The AI system analyzes all grayscale 2D ultrasound cines of a study and detects 8 morphological findings suspicious for CHD. The findings were selected such that most major forms of CHD would feature at least one such finding, and the presence of any of these would flag the study for referral for further examination.

We retrospectively included 877 exams (obstetric or detailed anatomic ultrasounds, or fetal echocardiograms), in singleton pregnancies, between 18‐24 weeks gestation from 11 centers, including 311 exams with a major CHD, cardiomegaly or levo/dextrocardia. Each exam included 4‐chamber, LVOT and RVOT views documented in cines, and was not used for training of the AI system.

For each exam, 3 expert fetal cardiologists annotated the presence or absence of each finding and majority voting was used as the ground truth. The AI system predicted the presence, absence or inconclusiveness (in case of low confidence) of each finding. An exam was predicted positive if at least one finding was present, negative if all findings were absent and inconclusive otherwise.

**Results**: Regarding the detection of any finding, the AI system had a conclusive output for 98.8% (95%CI 97.8‐99.3) of exams. On exams with a conclusive output, sensitivity was 98.7% (96.7‐99.5), and specificity was 97.7% (96.1‐98.6). Performance per individual finding is presented in the Figure. To better represent the general low risk population despite the artificially high prevalence of exams with at least one suspicious finding (311/877), per‐finding specificity was computed on exams negative to all findings.

**Conclusion**: The AI system accurately detected 2T ultrasound exams suspicious for CHD consistent with experts in most cases. The AI system may improve the prenatal ultrasound detection of CHD and thereby improve outcomes.

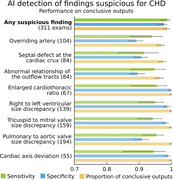

## 31 | Effect of Home Ultrasound in Patients with Previous Late Pregnancy Loss‐ A Randomized Control Trial

Liat Mor^1^; Hagit Eisenberg^2^; Liliya Tamayev^3^; Daniel Tairy^1^; Ben Oren^3^; Yael Ganor Paz^1^; Michal Levy^3^; Eran Weiner^2^; Giulia Barda^2^

*^1^Edith Wolfson Medical Center, Holon, HaMerkaz; ^2^Wolfson Medical Center, Wolfson Medical Center, Tel Aviv; ^3^Edith Wolfson Medical center, Holon, HaMerkaz*

2:00 PM ‐ 2:15 PM

**Objective**: We aimed to evaluate the effect of incorporating twice‐weekly telemedicine home‐ultrasound sessions on maternal anxiety and antenatal attachment in patients with a history of late pregnancy loss.

**Study Design**: In this randomized controlled trial, patients with a history a pregnancy loss beyond 20 weeks of gestation were recruited during their subsequent pregnancy and randomized into either a control group receiving standard high‐risk care or a study group receiving additional twice‐weekly home‐ultrasound sessions. Home‐ultrasound scans were guided by a physician via telemedicine and assessed basic fetal wellbeing using a Pulsenmore device. Two validated questionaries assessed maternal anxiety‐ the State‐Trait Anxiety Inventory Scale (STAI‐S) and the Revised Prenatal Distress Questionnaire (NuPDQ). Maternal attachment was evaluated using the Maternal Antenatal Attachment Scale (MAAS‐2). All questionnaires were filled in the beginning, mid and end of follow up time. The primary outcome was the STAI‐S score at the final prenatal visit (STAI‐3). In order to detect a 20% difference in the primary outcome, a sample size of 50 patients was required.

**Results**: Fifty patients were recruited and completed the study, 25 in each group. Demographic characteristics were similar between the groups. The home ultrasound group presented significantly decreased STAI‐3 scores (p = 0.022), a significantly increased MAAS‐3 score (p = 0.022) and less unscheduled emergency department visits during this time (p = 0.024) compared to control. Multivariate regression analyses confirmed significant and independent associations between reduced STAI and improved MAAS and the use of home‐ultrasound (Table 1).

**Conclusion**: Incorporating home‐ultrasound visits into prenatal care in patients with prior late pregnancy loss may significantly reduce maternal anxiety and enhance maternal attachment. These findings suggest that home ultrasound technology can be a valuable tool in managing maternal anxiety in this subset of patients.

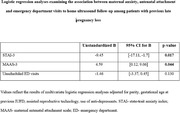

## 32 | Genetic Diagnoses in a National Cohort with Non‐Immune Hydrops and Other Fetal Effusions

Natalie B. Gulrajani^1^; Billie R. Lianoglou^2^; Katie Tick^3^; Nuriye Sahin‐Hodoglugil^3^; Ugur Hodoglugil^4^; Patrick Devine^4^; Jessica Van Ziffle^4^; Mary E. Norton^2^; Teresa N. Sparks^3^

*^1^University of California, San Francisco, School of Medicine, San Francisco, CA; ^2^Center for Maternal‐Fetal Precision Medicine, University of California San Francisco, San Francisco, CA; ^3^University of California, San Francisco, San Francisco, CA; ^4^Genomic Medicine Laboratory, University of California, San Francisco, San Francisco, CA*

2:15 PM ‐ 2:30 PM

**Objective**: Many single gene disorders are associated with non‐immune hydrops fetalis (NIHF) and other fetal effusions. However, less is known about how these diseases present uniquely in utero. We aimed to characterize how genetic diseases present in utero with NIHF, other single effusions, and concurrent anomalies.

**Study Design**: This is a prospective national cohort of pregnancies with unexplained NIHF, pleural effusion, pericardial effusion, ascites, skin edema, cystic hygroma, increased nuchal translucency (NT) ≥3.5mm, or a combination of these. All ultrasound reports for each pregnancy were reviewed. A CLIA‐approved laboratory performed exome sequencing (ES) and results were provided to patients. Each case was categorized as presenting with NIHF, single effusion (pleural or pericardial effusion, ascites, or skin edema), or increased NT or cystic hygroma, all with or without concurrent structural anomalies.

**Results**: 118 pregnancies underwent ES. There was a high diagnostic yield among cases with NIHF and single fetal effusions (17‐36%), both with and without concurrent anomalies (Table 1). For cases with increased NT or cystic hygroma, the diagnostic yield was 42% for cases with concurrent anomalies, but zero in the absence of concurrent anomalies. Genetic diseases identified varied widely (Fig. 1). RASopathies, musculoskeletal disorders, and cardiomyopathies presented variably with and without concurrent anomalies and with all types of fetal effusions. Primary lymphedema syndromes presented later in gestation with NIHF and without concurrent anomalies. Diseases associated primarily with postnatal neurodevelopmental delay were seen with single fetal effusions and increased NT, with and without concurrent anomalies.

**Conclusion**: ES yielded a diagnosis in a high proportion of cases with NIHF and other single fetal effusions, with the exception of isolated increased NT or cystic hygroma. Certain genetic diseases presented broadly with all types of fetal effusions, while others presented more uniquely. Understanding these patterns informs accurate prenatal diagnosis, clinical counseling, and management.

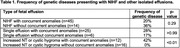

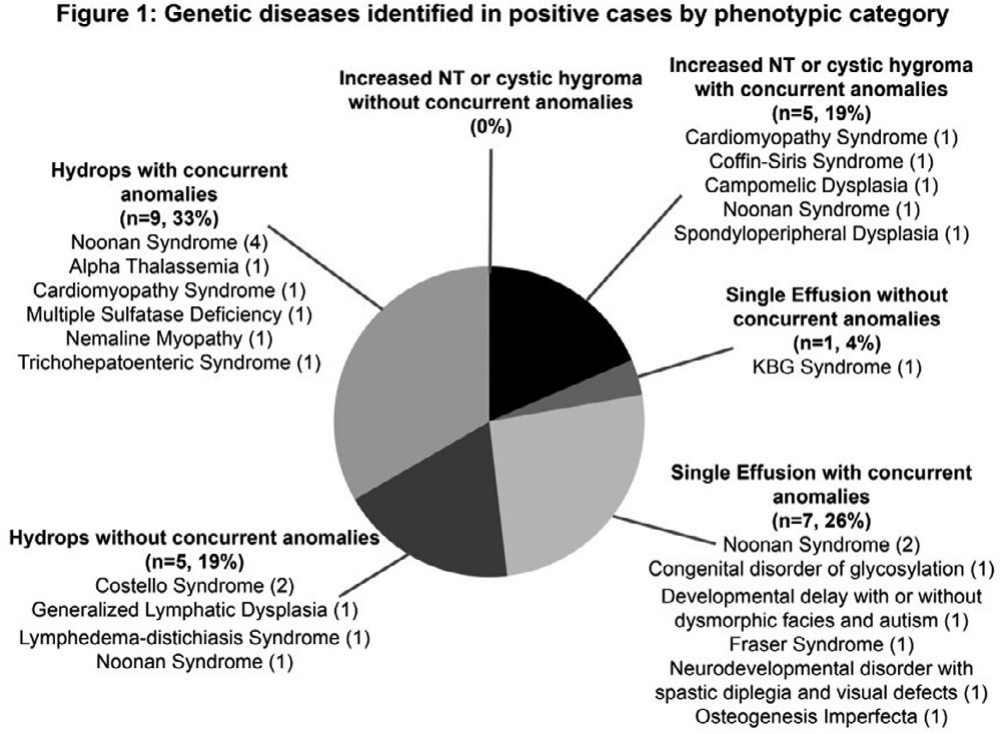

## 33 | Genomewide monogenic Non Invasive Prenatal Genetic Screening based on maternal cfDNA

Dolev Rahat^1^; Ravit Mesika^1^; Lilach Schneor^1^; Noa Liscovitch‐Brauer^2^; Tom Rabinowitz^1^; Noam Shomron^1^; Reut Tomashov Matar^3^; Lina Basel‐Salmon^3^

*^1^Identifai Genetics, Tel Aviv, Israel; ^2^Identifai Genetics, Tel Aviv; ^3^Raphael Recanati Genetic Institute, Rabin Medical Center, Beilinson Hospital, Tel Aviv*

2:30 PM ‐ 2:45 PM

**Objective**: Current methods for prenatal screening using cell‐free DNA (cfDNA) for monogenic disorders have significant limitations. They are restricted to a predefined set of variants or genes, require impractically high fetal fractions (FFs) for clinical settings, or focus on paternally‐inherited variants. Since paternal genetic material is often unavailable, there is a critical need for a father‐independent solution. We present a comprehensive noninvasive prenatal genetic screening method that accurately predicts the fetal genotype at any location in the genome using only a maternal DNA sample.

**Study Design**: We conducted a study involving nine families with singleton pregnancies, with varying FF values (7‐25%) where the parents are carriers for a severe monogenic disorder. We performed whole genome sequencing (WGS) of both maternal genomic DNA and maternal plasma and predicted the fetal genotypes for the pathogenic variants and across the entire genome.

**Results**: Our study includes seven families with a pathogenic single nucleotide variant (SNV): four families where both parents shared a common recessive pathogenic variant (e.g., congenital chloride diarrhea), two families with suspected compound heterozygous pathogenic variants (e.g., cystic fibrosis), and one family with a dominant pathogenic variant (Treacher collins syndrome). We accurately predicted the fetal disease status in all tested families (Table 1). We also conducted a genome‐scale analysis of our algorithm's performance over millions of genomic sites where the mother was a carrier. Our method achieved an area under the curve (AUC) of 0.89 for homozygous variants (Figure 1).

**Conclusion**: Our study presents a novel approach for genome‐wide non‐invasive prenatal screening, addressing a critical clinical need. We demonstrate the clinical feasibility of this approach as early as the first trimester. Our genome‐wide method can be extended to detect variants of all types and scales, including copy number variations and aneuploidies, and it also allows for the detection of de novo fetal variants.

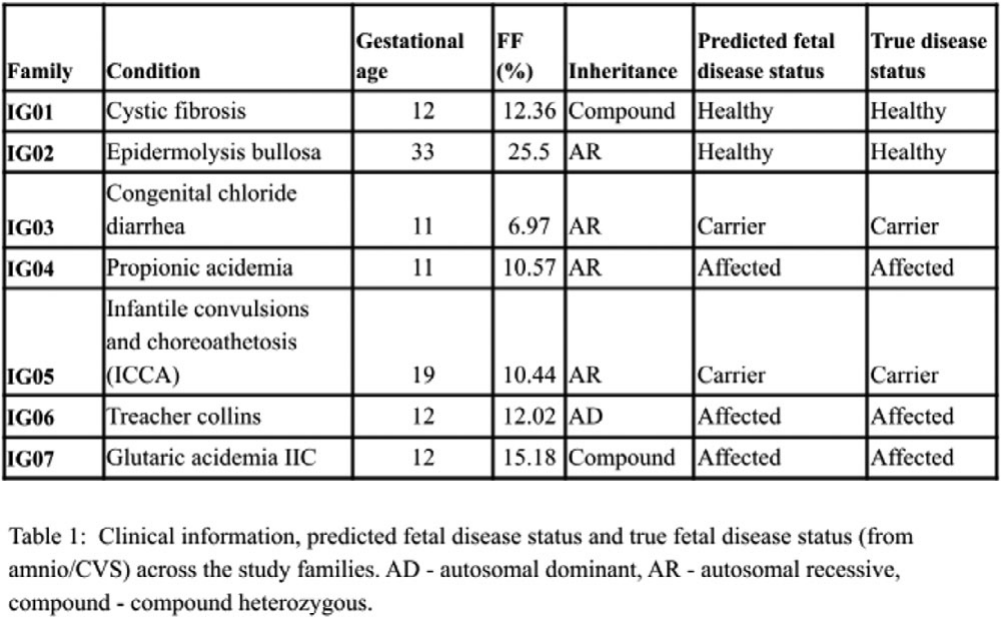

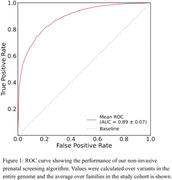

## 34 | AI Assistance Enhances Physician Performance in Identifying Congenital Malformations

Clémentine Morisset^1^; Frédéric Logé‐Munerel^1^; Celia Amabile^1^; Louis Chouinard^1^; Vianney Debavelaere^1^; Remi Besson^1^; Nikola Matevski^1^; Guillaume Corda^1^; Julien Stirnemann^2^; Yves Ville^3^; Yinka Oyelese^4^; Andrew Combs^5^

*^1^Sonio, Paris, Ile‐de‐France; ^2^Hôpital Necker Enfants Malades, Paris, Ile‐de‐France; ^3^University and Necker‐Enfants Malades Hospital, Paris, Ile‐de‐France; ^4^Beth Israel Deaconess Medical Center/Harvard Medical School, Boston, MA; ^5^Pediatrix Medical Group, Sunrise, FL*

2:45 PM ‐ 3:00 PM

**Objective**: Accurate sonographic identification of congenital malformations by interpreting Healthcare Practitioners (HCP) is critical for effective prenatal care. An AI software (under development, not FDA cleared) was trained to detect 10 fetal signs on automatically recognized views within its scope. This study aims to evaluate whether physician performance in identifying malformations improves with the assistance of the AI software versus without.

**Study Design**: A total of 1453 fetal images were labeled by experts and then reviewed by five physicians with varied profiles (Maternal‐Fetal Medicine specialists, Obstetricians/Gynecologists, and General Radiologists). The reviews were conducted in two sessions (with and without the AI software), separated by one week to minimize recall bias. Each provider reviewed a mix of pathological and non‐pathological images.

**Results**: There was a clear improvement for each reviewer and in overall performance ‐ from 40% sensitivity (92% specificity) to 70% (96% specificity) with AI support (Figure 1). When assisted by the AI, physicians refined their reviews to more closely match the AI's output, leading to notable enhancements in performance (Figure 2).

**Conclusion**: AI‐powered review of fetal images improves physician identification of signs of fetal malformations, highlighting the potential clinical impact of AI software to support more precise diagnosis.

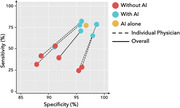

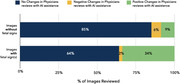

## 35 | Exploring Gastroschisis Patterns and Pesticide Use in California's Central Valley: A population‐based cohort Study

Alejandro Perez^1^; Bharti Garg^2^; Hope Delgado^3^; Joshua F. Robinson^4^; Aaron B. Caughey^2^; Stephanie L. Gaw^4^

*^1^University of California, San Francisco, Kerman, CA; ^2^Oregon Health & Science University, Portland, OR; ^3^University of California, Berkeley, Berkeley, CA; ^4^University of California, San Francisco, San Francisco, CA*

3:00 PM ‐ 3:15 PM

**Objective**: The incidence of gastroschisis has been rising in regions with significant agricultural practices. Certain pesticides (e.g. atrazine, mineral oil) have been associated with gastroschisis in epidemiological studies.

**Study Design**: This is a retrospective cohort study of all California births from 2016‐2020 utilizing linked birth certificate and hospital discharge records. We identified gastroschisis cases by ICD‐10 code Q79.3 and geographically assessed maternal residence by zip code. Pesticide usage by county was obtained from publicly available databases from California Department of Pesticide Regulation for years 2016‐2020. The usage of pesticides by county was calculated by pounds applied per total land area. We analyzed data from the following pesticides previously linked to gastroschisis: atrazine, mineral oil, oxyfluorfen, and glyphosate. We also investigated sulfur, which was the most widely applied pesticide in CA during this time period. Multivariable logistic regression, adjusting for maternal demographics, socioeconomic factors, and birth‐related variables was used to identify associations between pesticide application and gastroschisis.

**Results**: During the study time, there were 2,218,394 births and 656 cases of gastroschisis (0.3 cases/1000 births). Pregnant individuals in the San Joaquin Valley had 1.4 times higher odds of gastroschisis (95% CI: 1.1‐1.7) compared to other CA regions (0.5/1000 vs 0.3/1000, respectively). Analysis of the five pesticides of interest revealed higher gastroschisis rates in counties with greater usage of sulfur (aOR = 1.22; 95% CI: 1.00‐1.49; p = 0.046) and mineral oil (aOR = 1.24; 95% CI: 1.05‐1.45, p = 0.009).

**Conclusion**: These findings suggest an association between pesticide exposure, particularly sulfur and mineral oil, and risk of gastroschisis. More studies are needed to further illuminate causal relationships and guide targeted preventive strategies and management approaches for reducing gastroschisis incidence in vulnerable populations.

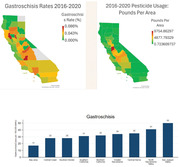

## 36 | AI Software Demonstrates Strong Performance for Screening of Tetralogy of Fallot and Truncus Arteriosus Communis

Remi Besson^1^; Nicolas Fries^2^; Julien Stirnemann^3^; Yves Ville^4^; Guy Vaksmann^5^

*^1^Sonio, Paris, Ile‐de‐France; ^2^Imagyn'Echo, Imagyn'echo Montpellier, Languedoc‐Roussillon; ^3^Hôpital Necker Enfants Malades, Paris, Ile‐de‐France; ^4^University and Necker‐Enfants Malades Hospital, Paris, Ile‐de‐France; ^5^Cabinet Vendôme, Lille, Nord‐Pas‐de‐Calais*

3:15 PM ‐ 3:30 PM

**Objective**: Congenital malformations are under‐diagnosed during fetal ultrasound (US) exams. Artificial Intelligence (AI) could make the examination safer by drawing the practitioners eyes to suspicious images. Objective is to evaluate the feasibility to build an AI recognizing the overriding great vessel images of the Tetralogy of Fallot (ToF) or Truncus arteriosus communis (TAC).

**Study Design**: An AI software (under development, not FDA approved) was trained on tens of thousands annotated US images from 30 major USA and European sites including the images of 102 patients with diagnosed ToF. No cases of TAC were used by the algorithm during the training phase. 

A first validation database is composed of 99 overriding aorta from ToF cases and 2153 normal images coming from these same centers. A second validation database was composed of 21 ToF and 7 TAC cases built from literature images between 2001 and 2021. Each exam was reviewed by one fetal US expert responsible for extracting all the images showing an overriding great vessel. The AI software was evaluated on its ability to accurately identify this sign.

**Results**: On the 99 overriding aorta images of the first validation database, the AI software reached 94.95% sensitivity in raising an alert. On the literature images, respectively on ToF and TAC cases, the algorithm identified an overriding great vessel on resp. 36 and 12 images, reaching a sensitivity of resp. 90% and 92.3%. On the control group, 98.8% specificity was reached (Fig.1).

**Conclusion**: This study demonstrated the feasibility in building a reliable alert system for screening of ToF along with the performance of such an algorithm in detecting pathologies non‐seen during training (TAC) thanks to including well‐known ultrasound semiology to guide the AI learning phase. Further tests on independent DBs from new centers are needed to better assess the AI software's robustness.

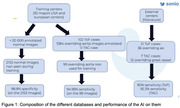

## 37 | Genetic Etiologies of Bilateral Renal Agenesis

Bobby Brar^1^; Robert Weatherford^2^; Carol Nowlen^1^; Katelynn Sagaser^3^; Ahmet A. Baschat^1^; Karin Blakemore^1^; Jena L. Miller^1^; Angie C. Jelin^1^

*^1^Johns Hopkins Medicine, Baltimore, MD; ^2^Massachusetts General Hospital, Boston, MA; ^3^23andMe, South San Fransisco, CA*

3:30 PM ‐ 3:45 PM

**Objective**: Bilateral renal agenesis (BRA) with anhydramnios due to fetal anuria has a poor prognosis due to severe pulmonary hypoplasia. Although often isolated, BRA may be one feature of a larger, complex genetic disorder. Our objective was to further elucidate the genetic etiologies of fetuses with BRA. 

**Study Design**: We performed a retrospective cohort study of all patients with fetal BRA presenting to an academic medical center for evaluation under the Renal Anhydramnios Fetal Therapy (RAFT) trial over a five‐year period. Maternal demographics, ultrasound reports, and genetic screening/diagnostic testing results were reviewed. Pedigrees were also assessed to help inform potential inheritance. 

**Results**: From 2017–2022, 52 patients with fetal BRA presented for evaluation. Extrarenal anomalies were seen in 17 (33%), most commonly cardiac (11), CNS (4), and skeletal (4). Aneuploidy screening was performed in 32 (62%), and none screened positive for the common autosomal aneuploidies. Diagnostic testing, offered to all, was completed in 37 patients (71%) with a 19% testing yield (7/37) (Table 1). 25 of the 37 had isolated BRA. Of 14 karyotypes, 1 case of 47, XXX was appreciated. Chromosomal microarray, performed in 28 patients, identified 3 copy number variants (CNVs): (2q21.1 duplication, 16p13.11 duplication, and 22q11.2 microdeletion). Exome sequencing revealed a pathogenic abnormality in 3 out of 5 patients (heterozygous variants in *GREB1L* and *RET*, compound heterozygous variants in *FREM2*). Of 7 patients with abnormal diagnostic testing, only 3 had extrarenal anomalies. Pedigrees were elicited in 46 patients with 13 (28%) reporting a family history of a genitourinary anomaly. 

**Conclusion**: Approximately 1 in 5 fetuses with BRA have abnormal genetic testing. This highlights the importance of a comprehensive diagnostic testing approach in this population. Future research routinely employing molecular methodologies that interrogate for potentially etiologic CNVs and single gene disorders in pregnancies with BRA is needed to further our understanding of this condition, and to better inform parental counseling.

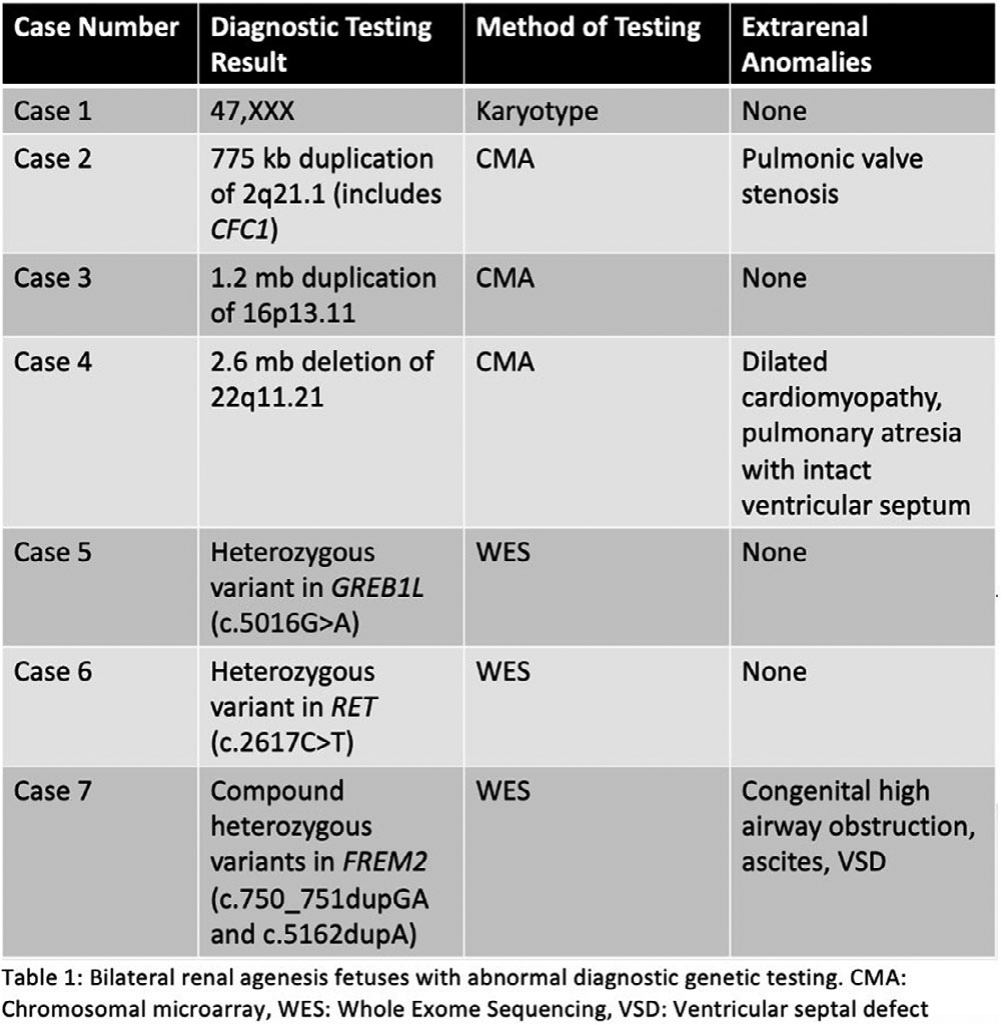

## 38 | Fully Quantitative Cervical Remodeling: Inter‐pregnancy interval shows differences in biomechanical characteristics of the cervix

Sarah M. Dwyer^1^; LeAnn A. Louis^1^; Methodius G. Tuuli^2^; Adam K. Lewkowitz^2^; Julie Tumbarello^1^; Emily Dively, BSN^3^; Madeline Felske^1^; Wendy Sparks^1^; Giselle Kolenic^4^; Peinan Zhao^5^; Molly J. Stout^6^

*^1^University of Michigan Hospital, Ann Arbor, MI; ^2^Women & Infants Hospital of Rhode Island and Alpert Medical School of Brown University, Providence, RI; ^3^Washington University School of Medicine, St. Louis, MO; ^4^University of Michigan, Ann Arbor, MI; ^5^Washington University, St. Louis, MO; ^6^University of Michigan Medical Center, Ann Arbor, MI*

3:45 PM ‐ 4:00 PM

**Objective**: Short interval pregnancy is known to confer obstetric risks in subsequent pregnancies. We aimed to examine starting cervical stiffness based on time elapsed since prior pregnancy and mode of delivery in prior pregnancy.

**Study Design**: We invented fully quantitative cervical elastography (FQ‐CES), which modifies a transvaginal ultrasound probe to quantify both pressure applied and tissue deformation, thereby yielding a fully quantified strain‐based elastography system that provides a numeric quantification (Young's modulus) of cervical tissue stiffness.  This system is operator independent and can be compared across patients and within patients over time.  This analysis is a prospective cohort study of people with singleton pregnancies presenting for prenatal care. Exposure is time in years since immediate prior pregnancy and mode of delivery of prior pregnancy. Outcome is fully quantitative cervical stiffness in the first trimester. Mixed regression models were used.

**Results**: 196 individuals included. Median years since prior pregnancy is 2 years (IQR 1,3 years). Prior vaginal birth shows a progressive hardening (13.8% change in Young's Modulus per year) over the 3 years following birth (p = 0.18) whereas cesarean birth shows stable cervical stiffness (1.3% change in Young's Modulus per year; p = 0.95).

**Conclusion**: Cervical tissue remodeling after pregnancy may be differ after vaginal versus cesarean births. First trimester cervical stiffness may be associated with time since last pregnancy. The inter‐pregnancy interval is a critically understudied time window which may provide insights into optimizing pregnancy outcomes.